# Comparing and Contrasting Knowledge on Mules and Hinnies as a Tool to Comprehend Their Behavior and Improve Their Welfare

**DOI:** 10.3390/ani9080488

**Published:** 2019-07-26

**Authors:** Amy McLean, Angela Varnum, Ahmed Ali, Camie Heleski, Francisco Javier Navas González

**Affiliations:** 1 Department of Animal Science, University of California-Davis, Davis, CA 95616, USA; 2 College of Veterinary Medicine and Biomedical Sciences, Colorado State University, Fort Collins, CO 80523, USA; 3 Department of Animal Science, Michigan State University, East Lansing, MI 48824, USA; 4 Department of Animal and Veterinary Sciences, Clemson University, Clemson, SC 29634, USA; 5 Department of Animal and Food Science, University of Kentucky, Lexington, KY 40506, USA; 6 Department of Genetics, Veterinary Sciences, University of Cordoba, 14071 Córdoba, Spain

**Keywords:** mules, hinnies, behavior, aggression, early foal handling, welfare

## Abstract

**Simple Summary:**

Mules and hinnies combine traits of their equid parents—the horse and donkey—but are less studied or understood. Still, their welfare varies greatly because of several factors. These hybrids have anatomy, health, nutritional, and behavioral particularities that are distinct from those of donkeys or horses. Their behavior can pose challenges to providing routine care and treatment during times of disease. Abusive treatment can result from those who have little understanding of learning theory or body language. Hence, an overview of studies and field observations can offer solutions for welfare enhancement. According to literature, participatory surveys and behavioral assessments across several countries, mule owners and handlers find it easier to interact with their animal as compared to allowing a stranger to do so. By contrast, unfamiliar persons had more success with mules handled at an early age. Gaining trust and proceeding with slow and methodical movements tend to work best for working with mules and hinnies. Early foal handling additionally creates success in training and behavior modification. Conclusively, the key to improving mule and hinny welfare is to shape human behavior, creating a training approach which will ultimately improve the animals’ welfare.

**Abstract:**

Mules and hinnies are the hybrids between donkeys (*Equus asinus*) and horses (*Equus caballus*). For centuries, mankind has used them for agrarian purposes, the military, or recreation. Contrasting literature with behavioral observations, we seek a better behavioral understanding andthus comprehensive solutions for their welfare enhancement. Over the past 6 years, we have assessed physical and behavioral welfare in over 900 mules by surveying owners from Egypt, Peru, Portugal, Spain, Mexico, and the U.S. These mules participated in shows, brick kiln work, cart-pulling, packing, tourism, and cattle herding. Observations are discussed alongside facts from the literature. Unfortunately, their behavior has been misunderstood by many, and harsh treatment and equipment has been used to control them. Few studies have attempted to define or use learning theory to understand how and why mules and hinnies behave as they do. Hence, understanding their health considerations, natural behavior, and training theory is crucial for those who work with them.Solutions to welfare improvement partially lie in an individual’s ability to handle mules and hinnies from birth, and to proceed slowly through training. Conclusively, this review sets forth a clearer understanding of these hybrids’ behaviors and promotes positive handling, allowing their access to more routine healthcare and ultimately, a higher welfare standard.

## 1. Introduction

**Figure 1:**
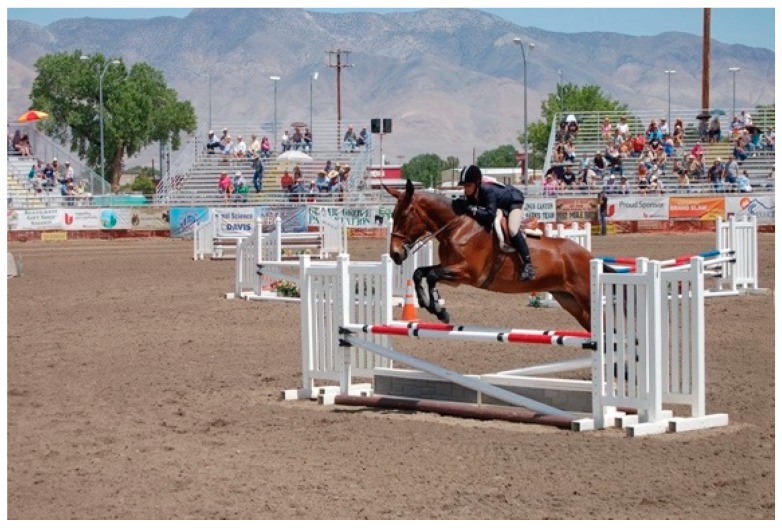
Mules and hinnies can be trained to compete in multiple disciplines, just like horses. The U.S. has seen a growth in mules, hinnies, and donkeys for recreation and competition mounts.

The Equus family includes horses, donkeys, mules, and hinnies. Globally, there are 14 million mules and hinnies—the smallest sector of the equid population (112 million, 50 million horses, and 54 million donkeys) [1]. A mule is the cross between a male donkey, a jack, and a female horse, a mare. The reciprocal cross produces a hinny. They serve important roles as working equids generating critical income and have grown in popularity amongst horse owners looking for recreation and competition mounts worldwide [2,3,4]. Mules can be trained to compete in multiple disciplines, from dressage, jumping, Western riding, reining, and racing [5] (Figure 1). In the U.S., the mule and donkey population increased from 2007 to 2012 even as the number of U.S. horses and ponies declined [6].

Despite their prevalence, mules and hinnies are some of the least-studied equids alive. Due to the increased number of hybrid equids (crosses between different equine species) owned for recreational purposes, the professional community has seen an increased demand for information on mules and hinnies [7]. The donkey is often thought of as the least researched member of the equids, but even less is known about mules and hinnies [8]. Mules and hinnies have proven to be more challenging to research based on their genetic variation, especially from the horse side. Over the past ten years, there has been an increase in the number of research articles and information for donkeys. Such a growth in available information has yet to be seen for mules, and even less so for hinnies, but this review compares some of the most relevant contextual and global information available from multiple countries and behavioral assessments [9,10].

**Figure 2:**
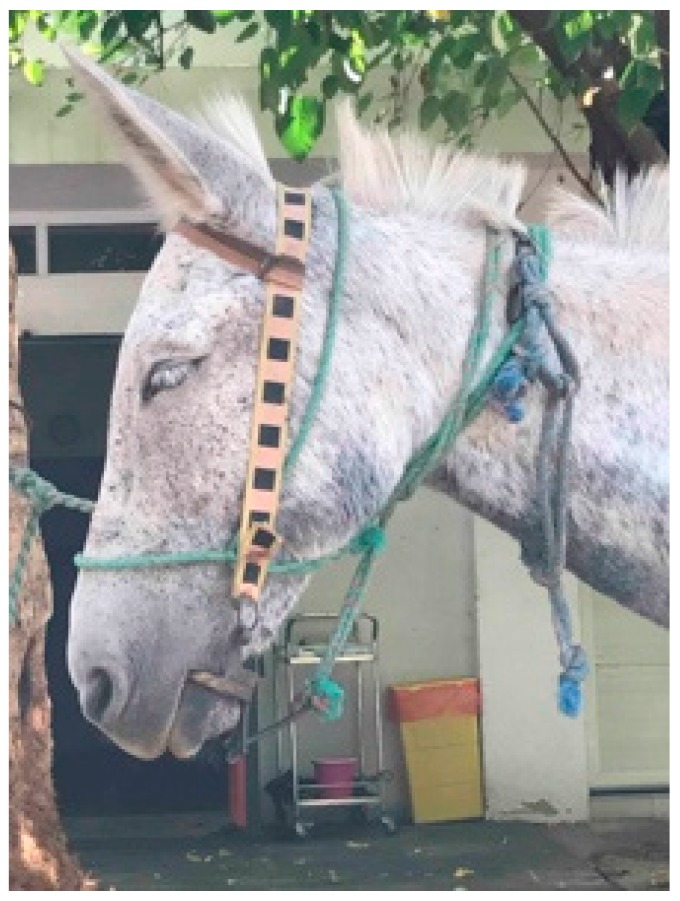
Mules and hinnies are often worked or ridden with harsh equipment to control their behavior such as this ring bit. This can result in lacerated tongues and/or paralysis to the tongue. Improved understanding about learning theory could help reduce the use of such abusive equipment.

**Figure 3:**
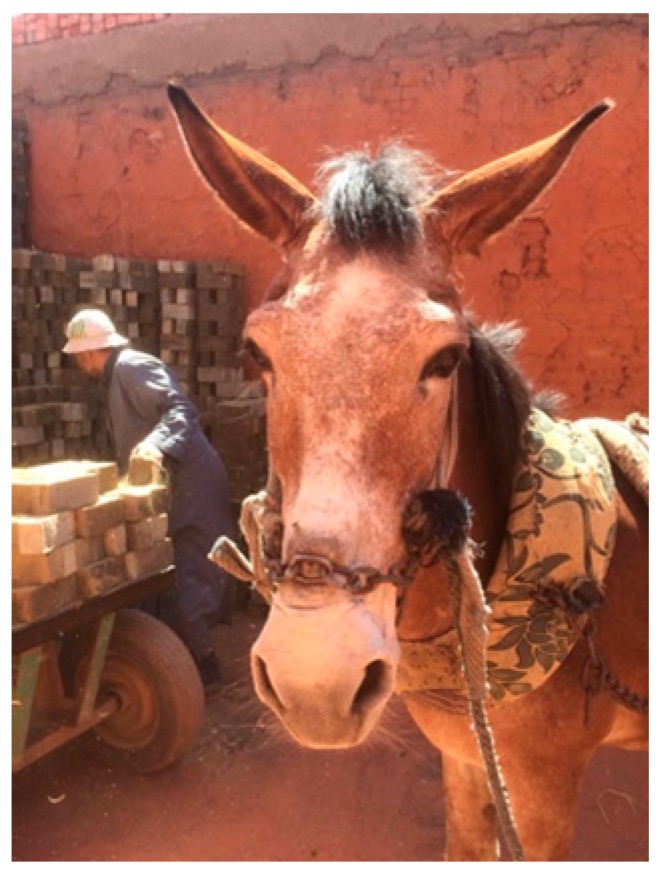
Another example of harsh equipment being used to control a mule working in an Egyptian brick kiln. The chain is purposefully used to create lesions on the nose to sensitize the mule to further pressure. Improved understanding of mule behavior would decrease the need for abusive tools such as this.

A new mule owner may first wonder which parent the animal should be compared to, the horse or the donkey. A handler or owner of a mule or hinny in a working capacity may question why this animal is so difficult to handle and train. Some of the greatest compromises in welfare for mules and donkeys are due to a lack of understanding of their behavior. There are many fallacies and opinions related to working with, treating, training, and owning mules and hinnies, especially when comparing them to horses (Figure 2 and Figure 3).

Some experts will agree that mules are not for everyone [8,9]. The term “muleskinner” has been applied to a person who can understand the ways of a mule and work around the mule’s peculiarities without injury [8]. Mules have outperformed both parents, and even dogs, on cognition tests [11,12], showing that hybrid vigor may even enhance cognitive function [13]. Often, routine procedures with mules require more patience and work than when working with a horse [7,8]. One of the greatest assets when working with mules is working with someone who is familiar with the mule and its behavior. It is not uncommon for mules to exhibit signs of avoidance or fearfulness when an unfamiliar person makes repeated attempts to approach the animal for routine procedures or husbandry tasks (e.g., administering injections, taking a rectal temperature, or picking up a hoof). Several studies have concluded this by comparingmule responses during approach tests conducted by familiar and unfamiliar people [14,15,16]. To the knowledge of the authors, the same information is not available on hinnies in the literature.

Mule behavior has been incorrectly perceived by many; even more remarkably, the lack of behavioral contrasted information about hinnies only worsens this knowledge gap. A better understanding of the behavior of both hybrids could lead to improved welfare [13,15]. The objective of this work is to provide an overview of studies and behavioral observations related to mules and hinnies, review participatory approaches to understanding their behavior, relate research results to years of observations in the field, and offer solutions for welfare enhancement based on behavioral understanding.

## 2. Behavior and Welfare

### 2.1. Attitudes Toward Mules and Hinnies

The reason that some owners choose mules instead of hinnies, or donkeys instead of horses, is often due to resources, perceptions about intelligence, attitudes, and strengths. Some authors have suggested that hinnies are more docile when compared to mules due to inheriting the donkey temperament [17]. Given the higher costs of mares when compared to female donkeys (jennies), in some impoverished areas the availability of mares is very limited and jennies are more readily available, so hinnies are produced. During Roman times, hinnies were preferred partly due to the availability of jennies as compared to mares [18,19]. In addition, Celtic communities reported having large populations of hinnies compared to mules which could be representative of the number of jennies versus mares that were available for breeding [18,19]. In Celtic communities, hinnies were reported to be used as primarily war animals and mules were used in labor or draft roles [18]. Although the Egyptians were well known for producing mules for agricultural purposes and hinnies for pulling chariots, there is little reference in historical sources about either hybrid [20].

In a study comparing performance of working horses, donkeys and mules/hinnies in extreme weather conditions in Egypt, mules and donkeys showed fewer signs of heat stress, such as elevated rectal temperatures, than horses performing the same job and under the same weather conditions [21]. In another study comparing mules/hinnies and donkeys working in the Egyptian brick kilns, mules fared better than donkeys, which experienced considerably aggravated signs of heat stress [22].

Some people prefer mules to hinnies and associate hinnies with misconceptions, such as having a smaller stature than mules or inferior conformation (longer backs). For example, in 1834, French hippologist Louis-Furcy Grognier [23] reported that hinnies were seldom larger than their dam (the jenny), often smaller, and forms were viler: heads were narrower and longer; ears were shorter and more misplaced; necklines were thinner, with a more arched back and a sharper rump; legs were more proportioned; tail was more adorned with hair; and it neighed like the horse, instead of braying like the donkey or mule. Grognier suggested the hinny’s vocal characteristics and abundance of hair in their mane and tail may be inherited results from the stallion, whereastheir conformation is from the jenny [23]. Other arguments suggested the hinny was inferior to the mule regarding its size and strength [24]. Male hinnies and female mules were preferred over the opposite by those working the field because of their better abilities and nobleness [25]. Hinnies today may be favored due to their remarkable adaptability in extreme desert environments, more akin to their donkey relatives than their mule equivalents. In Baja, Mexico, hinnies have been used for over 300 years [14]. Hinnies and mules closely resemble their parents and come in all sizes and various conformations, reflecting characteristics of both their sires and dams.

**Figure 4:**
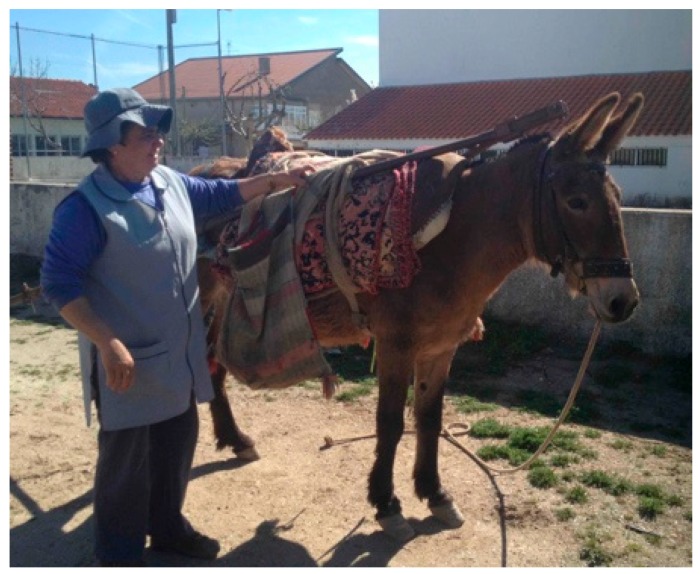
Hinnies are commonly found in many countries where access to mares has been traditionally limited. This particular hinny is used to plow and dig up potatoes and assist with harvesting grapes in Portugal. In some places they are preferred over mules.

To understand preferences for breeding hinnies or owning/working/showing hinnies compared to mules, horses, or donkeys, the authors asked those who are actively breeding and using hinnies to complete a survey. The surveys were conducted with hinny breeders and owners in Colombia, Mexico, Portugal, United States and Spain. The results suggested that hinnies were very sturdy animals, similar to mules, but inherited traits that closely resembled the stallion (e.g., gait of Paso Fino hinnies in Colombia, or conformation of superior Quarter Horses for show hinnies in the U.S.) [14,15,26] (Figure 4).

The use, and therefore training, of mules and hinnies reflects economic, geographic, and cultural trends, and can be wrapped in long-held cultural beliefs [10,14,15,26].

### 2.2. Behavioral Genetics

Mendelian predictions may state we should obtain the same product whether it is from breeding a jack to a mare or a horse to a jenny; however, the reciprocal cross yields a phenotypically different offspring [27]. The crossing of two equid species results in a hybrid with differences ranging from anatomical features, or phenotype, to behavior, togenetics (specifically, chromosome numbers). The diploid number of chromosomes is 64 for horses, 62 for donkeys, and 63 for mules and hinnies, respectively. In addition to the numerical differences between horse and donkey, there exist structural differences which are most noticeable as greater numbers of metacentric chromosomes in the donkey.

The first references stating a difference between mules and hinnies date back to the first century AC with Marcus Terentius Varro (rust. 2, 8, 1) and Columella (6, 37, 5) among others, who described a “mulus” (Latin for mule) which was the offspring of a mare and a jack and a “burdo” or “hinnulus” (Latin for hinny) which was an offspring of a male horse and a jenny. Apart from their different genetic basis, these equid hybrids have anatomy, health, nutritional needs, or behavioral particularities, which are distinct from those of donkeys or horses. When comparing the phenotype of donkeys, hinnies and mules, hinnies and donkeys were found to have similar balance when measuring the underline and topline. The mule was found to have closer to a 2:1 ratio seen in a well-balanced horse, meaning the underline is twice as long as the topline (back and loin) [28]. It has been widely reported that mules and hinnies may be a case of an ancestral epigenetic phenomenon called genomic imprinting. The phenomenon of genomic imprinting happens when the expression of a gene may be determined by its origin rather than its DNA sequence. Accordingly, hybrids (both mule and hinnies) may reciprocally differ, not only in their evident phenotypical conformational manifestations, but also in the behavioral responses associated with divergent molecular mechanisms (endocrine behavioral regulation), depending on whether the sire is a donkey and dam is a mare or sire is a horse and dam is a jenny [29,30].

The improved stamina and cognitive capabilities [13] of mules and hinnies compared to their parental species is one example of the occurrence of hybrid vigor; however, it is interesting that data on the interbreeding of the female donkey with the domestic horse are less readily available. Hinny production does not appear to have had any great economic importance [31]. In studies it has been shown that the genotype of the fetus is directly related to the level of gonadotrophic hormones in the maternal sera of the horse and donkey. McGovern [32] suggested that pregnant mare serum gonadotrophin (PMSG) fails to reach normal levels in mares carrying mule fetuses while the level of PMSG in a donkey carrying a hinny fetus was considerably higher than the levels which occur in donkeys during normal pregnancy. According to Allen [33], histological examination of endometrial cup tissue recovered from horses and donkeys carrying inter and intraspecific conceptuses reveals strong evidence of maternal cell-mediated immune reaction against the endometrial cup cells since PMSG remains detectable beyond 200 days of gestation in females mated to their co-twin brothers to which they exhibit skin graft tolerance. Commencing immediately after initial invasion of the chorionic girdle cells to form the cups and increasing steadily over the next 80 to 100 days, small lymphocytes, and later on also plasma cells and eosinophils, accumulate in the endometrial stroma surrounding the cup tissue [33]. In normal intraspecific pregnancies these aggregated cells remain clustered at the periphery of the cup tissue and they only begin to invade the cup itself after day 80–90 [33]. Then serum PMSG levels are declining and the large cup cell at the luminal surface of the cup have already begun to degenerate and slough off. In interspecific pregnancies, on the other hand, the leucocytic reaction occurs much more rapidly and is considerably more intense and the lymphocytes, instead of remaining clustered in the surrounding stroma, immediately begin to actively invade the cup tissue and attack the large cells [33]. Hence, the whole structure is prematurely destroyed with in as little as 10–15 days, and the necrotic tissue is sloughed off the endometrial surface by day 70. From these marked differences between the endometrial cups in intra and interspecific pregnancies it may be concluded that the mare or jenny recognizes paternal histocompatibility antigens on the invaded endometrial cup cells, in response to which she mounts a classical cell-mediated immune reaction, shortening the lifespan of endometrial cups [33].

### 2.3. Behavior Related to Anatomy, Health, and Nutrition

Mules and hinnies vary substantially in size, conformation, and disposition, making research design and universal guidelines about their care, challenging [8]. The common misconceptions about these hybrids and the lack of scientific literature were motives for this review’s aim, which is to highlight the differences setting mules and hinnies apart. When considering mules and hinnies as compared to donkeys, one must not forget their anatomical differences. The vocal folds of the donkey’s laryngeal anatomy yield the bray instead of a nicker or whinny [8]. Donkeys possess a difference in the opening of the guttural pouches, and the angle of airway varies from that of the horse; we would expect this to be true for mules and hinnies considering that their form of verbal communication is a combination of both a neigh and a bray. The conformation of mules and hinnies varies according to the breed of horse and donkey. Therefore, saddle and tack fit for mules and hinnies can be confounded by varying wither conformation depending on genetics: donkeys have less prominent withers but a more prominent sternum, and some mules may take on these characteristics. Tack used for mules may require breeching or a tail crupper to ensure proper fit. Mule hooves are small and boxy, a cross between those of horses and the thick sole and tough wall of donkeys [34]. Diagnostic results using hoof testers in mules, and especially donkeys, should thus by carefully interpreted [7,35].

#### 2.3.1. Health and Behavior

Perceptions associated with mule and hinny health are widely anecdotal and often incorrect. A basic part of a routine clinical exam may include evaluating blood cells and biochemical parameters, but little scientific information is available for mules and hinnies. One study focused on a healthy population of mules and known hinnies with the same genetics to define reference values for blood chemistry and hematology [36]. The study found significant differences (*p* = 0.005) in resting temperature between hinnies and mules, with the hinny’s temperature closest to that of the donkey, 37.06 °C ± 0.46 °C, and the horse and mule most similar at 37.24 °C ± 0.55 °C [36]. A significant difference was reported for red blood cells, packed cell volume, hemoglobin, mean cell volume, and mean cell hemoglobin [36]. Differences in biochemical parameters, liver enzymes, and white cells were found as well when comparing mules and hinnies to horses and donkeys [36]. Prior to this study such basic information was not and is currently not readily available for owners or practitioners, which can ultimately affect treatment and diagnosis and overall welfare of mules and hinnies.

Perceptions that all mules are naturally aggressive are common among younger mule handlers or those with less experience [10], but this is not always true. It can become nearly impossible to work with poorly-handled mules to perform the most basic husbandry or veterinary procedures. A recent study investigated whether mule aggression consists of innate acts toward human beings or is a reactive response to the rough handling procedures performed by handlers [29]. In that study more than ½ of the 374 handlers confirmed they were previously kicked or bitten by mules [29]. Approach tests with mules working in 374 brick kilns have shown that mules are more likely to exhibit signs of aggression (e.g., bite threat) towards or when approached by an unfamiliar person and are less likely to exhibit such behavior towards a familiar person [29]. This behavior was recorded fewer times in horses and the least amount of time in donkeys [28]. In that study, Ali and colleagues attempted to track the nature of aggression in mules and the risk factors that trigger such responses in mules found working in brick kilns. Over 370 mules from 50 different brick kilns showed signs of aggression 30% of the time during approach tests by an unfamiliar person, and 79% of the participants believed mules are inherently aggressive [29]. Mules in this study were found to have 42% of body lesions resulting from mistreatment, and significant correlations were found between the mules’ aggression and the handler’s attitude towards mules, age, and level of experience [29].

Behavioral misconceptions can additionally confound the physical examination and overall health assessment of mules and hinnies. For instance, some believe mules and hinnies will not colic or develop laminitis, among other conditions. However, they can overeat, and develop colic, laminitis, or metabolic disorders, just like horses do [7,8]. Rather, it is more likely that the pain associated with these clinical conditions may be less recognized in mules because of their stoicism [35]. Veterinarians, owners, and handlers who are familiar with mule and hinny behavior and variability in physical parameters can be instrumental in rapid diagnosis of pain or disease, and correct assessments of health, of these species [37].

#### 2.3.2. Nutrition and Feeding Behavior

Donkeys are considered to be browsers and horses, grazers. Mules and hinnies tend to perform a combination of both browsing and grazing. Mules and hinnies tend to consume forages high in fiber and require less grazing than horses [36]. Both mules and hinnies are found living and surviving on poor quality forages and in extreme environments [36], suggesting that they tend to be more like donkeys in terms of digestive efficiency (e.g., longer gastrointestinal transit time, large bites, and possible increased potential to recycle urea). Currently, no feeding guidelines are available for mules, although such guidelines exist for donkeys and wild equids in captivity [38]. It has been suggested that their body weight can be approximated using heart girth measurement tapes such as those intended for horses [39]. In addition to vigilant weight observations and recordings, routine body condition scoring of mules should be instituted to prevent overfeeding and obesity. Even though adipose tissue and deposition has been reported to be similar in donkeys and mules, fat deposition in donkeys mostly takes place along the crest of the neck, covering the ribs and croup areas. Furthermore, although it is common to see fat pones or adipose tissue in the croup and rump region in donkeys, in mules this appears to be the last place of deposition as noted in horses [36,40]. This supports the recommendation that the entire body of mules or hinnies be evaluated when evaluating body condition score; hands-on evaluation is necessitated.

### 2.4. Social Behavior

Mules tend to form strong bonds with other equids, especially other mules and sometimes horses [16,31]. When comparing dominance hierarchy among ponies, mules, and donkeys, one study found that ponies were most dominant, followed by mules, then donkeys. Ponies’, mules’, and donkeys’ rank was not correlated with age or height, but equid type. Mules preferred to stay with mules or ponies, and hinnies preferred to stay with hinnies or donkeys [16]. It has been suggested that mule and hinny foals form a stronger attachment with, or fondness of, their female ancestors, that is mules to mares and hinnies to jennies [31].

When confining mules and hinnies to stall or paddock areas, behavioral observations have suggested both may prefer a more open space versus confinement [41]. Stereotypies such as weaving or stall walking may be seen when mules and hinnies are isolated to a stall. Behaviors such as crib biting have not been recorded in mules or hinnies [41]. Both animals are very social and prefer buddies even in confined areas, such as pairing mules in a stall. Owners may find mules to display some behaviors that more or less need to be dealt with or accepted versus corrected (e.g., ear shyness, hard to catch, etc.) [42].

Studies have reported early embryonic loss in jennies carrying hinnies as well as low progesterone production, and differences in mating behavior [43,44]. Some have suggested that hinnies are harder to handle than mules, which may be related to the lower level of progesterone [45]. The role of progesterone levels on coping strategies and avoidance behaviors has been reported by several authors [45,46,47]. Particularly, active coping may be associated with lower basal corticosterone and progesterone [45]. Active coping strategies are aimed at the removal of the individuals from the source of stress or at removal of the stress source itself [45]. Interestingly, actively coping individuals have also been found to be more aggressive, and some have claimed the hinny inherits the docile nature of the donkey [27,45]. Further studies should be done in mules and hinnies focused on temperament and level of aggression.

### 2.5. Cognitive Abilities, Training and Behavior Modification

Mules and hinnies are not for everyone: it takes a very patient person to work with them [7,8]. Mules have proven to have a higher reasoning ability when compared to ponies and horses [16]. Mules, donkeys, and horses are used in recreation and sport, and are most often used for traction and transportation of goods around the world. In all situations, equids are usually worked in close contact with humans. An understanding of mules’ and hinnies’ cognitive abilities with a focus on interspecies variations will improve their welfare through adapted expectations by their trainers [12].

For instance, Osthaus et al. [12], in their study comparing the interspecies cognitive abilities in equids through testing the spatial abilities and preservation behavior of horses, donkeys, and mules in a detour task, reported that mules were fastest at solving this novel detour [12]. Moreover, mules were even faster than horses and donkeys in solving the repeated detour test [12] which suggests that mules may also be more flexible in modifying their behavior regarding spatial learning cognitive tasks than horses and donkeys. Such interspecies differences suggest that the hybrid vigor seen in mules might extend to their spatial cognitive abilities;far from being stubborn, they may be more flexible learners than horses and donkeys. If this was the case, we could hypothesize mules and hinnies would possess higher performance related to other cognitive processes and enhanced social learning abilities, which might contribute to them being even more favorable to train. However, no further interspecific study has been carried out to the knowledge of the authors.

**Figure 5:**
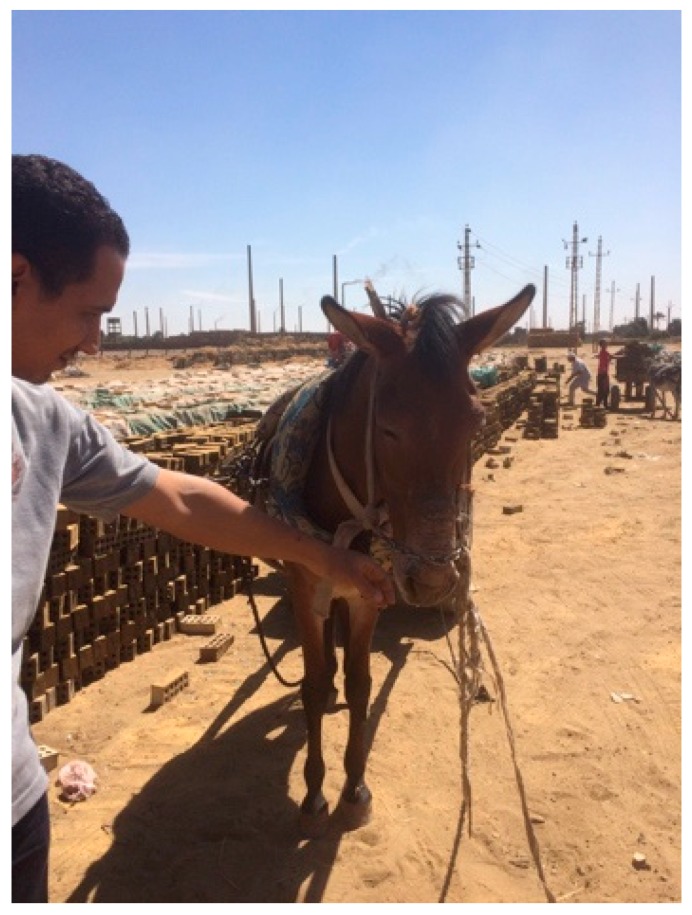
Approach tests in six countries have shown that mules are more accepting of familiar versus unfamiliar people. This makes attempting routine veterinary or husbandry procedures more challenging when treatment is being provided by an unfamiliar person [14,16].

Moreover, such interspecies differences between mules, donkeys and horses may extend beyond the context of spatial cognitive abilities to traits such as trainability and tolerance to humans in either an approach or a contact test by familiar/unfamiliar handlers. Working mules and hinnies that were used to pull loads were less tolerant of an approach by unfamiliar handlers and showed more trust to familiar handlers [10,15,16,32] (Figure 5). Pritchard et al. [16] found that mules being rented for riding purposes in India were likely to be more fearful due to inappropriate handling and compromised welfare. When the animal is rented versus owned, the bond or trust with the owner and mule is likely compromised and the new handler may use harsher methods of handling the mule out of fear [16]. Similar behavior observations have been noted when unfamiliar people are interacting with other animals [16].

In a study conducted in Mexico, donkeys tended to be most tolerant of unfamiliar persons touching their ears (100%, F_2.95_ = 32.3, *p*-value = 0.08) compared to mules (28%) or horses (17%) [14]. A study in Peru found that owners agreed it was easier for them to work with mules as compared to unfamiliar handlers (*p*-value = 0.001) [10]. When unfamiliar persons attempted to approach mules in Peru (*n* = 48) they were less successful than familiar persons. More familiar persons were allowed by mules to make forehead contact (38) as compared to unfamiliar persons (23), neck approach (34 compared to 0), and ear touch (28 compared to 0) [10]. The owners or handlers of these same animals had previously confirmed that it was easier for them to interact with their mules or hinnies as compared to unfamiliar people. In Egypt, we found mules would more likely allow familiar people to make chin contact (66% of the cases) compared to unfamiliar persons (16% of the cases). In the rest of the cases (18%) mules did not allow people to make contact whether it was by familiar or unfamiliar people. In addition, the mule was more likely to display signs of aggression toward, and overall avoidance of, the unfamiliar person (*n* = 410, 20, and 39%) [48]. Based on the display of avoidance (attempts to flee or move away) or aggression (attempts to bite, kick, and strike/paw), such behavior could be signs of defense ormisunderstanding, or due to poor, abusive, or a lack of handling. A higher proportion of donkeys showed avoidance (50%) than that of mules, while the least number of donkeys (10%) showed a friendly approach, as compared to 42% in mules. When working with mules or hinnies, observing and reading body language from the posture of the head, eyes, nostrils, neck, body, tail, and hind limb weight distribution will help one understand or predict a reaction.

All participants of surveys conducted in several countries among mule and hinny owners and handlers indicated that mules and hinnies must be handled from an early age. Mule and hinny breeders should ideally begin desensitizing their animals from birth, interacting with the foal by scratching, petting, and rubbing it from its ears to its tail. Picking up its hooves and working with its legs will help prevent difficult hoof trimmings. Some authors have reported that it is important to interfere as little as possible with the foal during the establishment of the mare–foal bond in horses [49], given neonatal handling could be counterproductive and affect bonding and social development negatively. Yet building a positive relationship with mules from day one tends to last a lifetime, evident at mobile clinics where young mules and hinnies from owners who began working with them from day one had superior behavior (Supplementary Materials Video S1). Mules and hinnies with such training tend to allow unfamiliar people to more easily approach them and provide treatment [15,27]. Other studies have suggested that foals should be handled early in life by the first week or two and at this time, they should be taught to lead, tie, have their feet handled, and be groomed [15,27,50,51,52].

Aggressive or challenging behavior from mules is a result of poor handling or violent handling and training [32,53] rather than an inner characteristic of the hybrid type. Other studies have reported similar findings and suggest that assessing the quality of human–animal (mule) bond is an essential part of welfare interventions for such populations [16]. Based on the authors’ experience and observations working with mules and hinnies, we have found that both respond well to positive reinforcers such as food rewards, tactile contact such as petting and scratching the neck and the inside of the ears, and verbal commands just like their donkey counterparts [54]. Mules tend to be more at ease when verbally provided commands versus no sound at all. When first approaching mules, it is often easier to approach their face versus their neck, where they will flee the situation [10,32] (Figure 4).

### 2.6. Methods of Restraining Behavior

**Figure 6:**
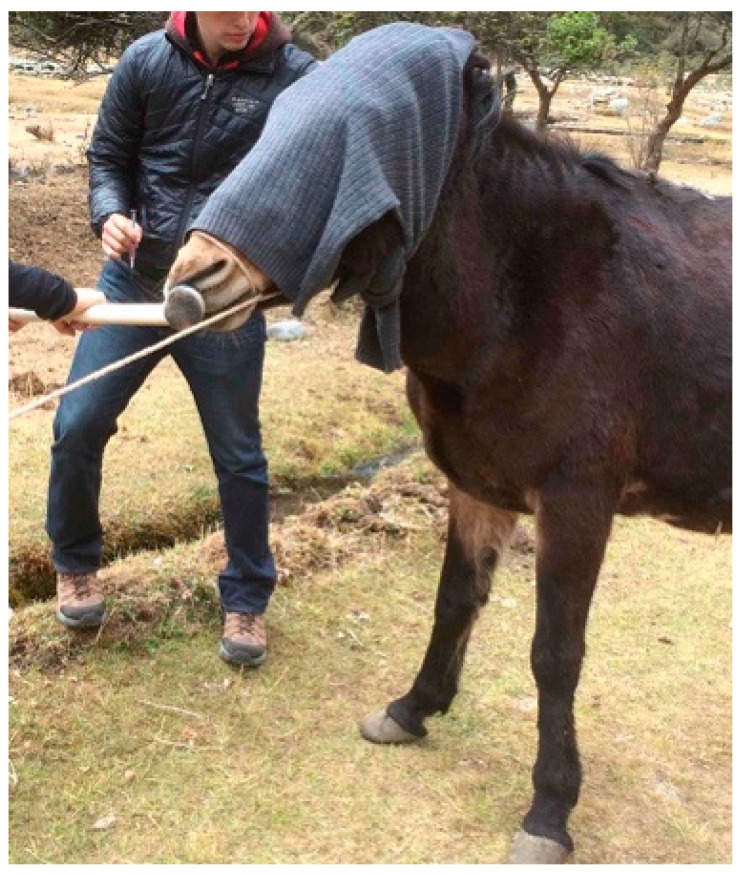
Judicious use of restraints may need to be applied to a fearful mule or hinny when some routine procedures such as administering an injection are performed. A blindfold, possibly in combination with a nose twitch, can help achieve calm administration of a vaccine and create less stress. This does not imply that using restraints is preferable to slow, careful training in more ideal circumstances.

A variety of methods to communicate with mules and hinnies are available, such as bits/bridles, halters/head collars with a variety of nose bands, materials, and widths [32,55,56,57]. Multiple restraint mechanisms with regional application have been noted as well, such as blindfolds, tying up legs, ear and nose twitches [15,39,56] (Figure 6). If a routine health task cannot be safely performed, nose twitches are a successful form of restraint, especially a string twitch with a long handle to hold (Figure 6). The restraint may actually decrease the stress placed on the mule/hinny when it is strongly resisting. As quoted by Fowler [58], the cause for this stress reduction relies on the fact that endorphins are released, lowering the heart rate and increasing tolerance for discomfort associated with procedures performed elsewhere on the limbs or body. Blindfolds used to cover mules’ or hinnies’ eyes are effective methods of calming an animal to receive treatment, including deworming or farrier work (Figure 6).

The application of these behavior restraining methods has raised welfare concerns within the scientific community. There are two different positions regarding their application in horses: On the one hand, there are researchers that suggest twitching the lip is an approach that should be adopted only when chemical restraint is not available; without giving full credit to the release ofbeta-endorphins or their effects on behavior, they argue the twitch works because it hurts [59]. The mechanism behind twitching has been discussed in many research papers. Resting plasma concentrations of β-endorphin concentration in adult horses was 22.4 ± 2.8 pmol mL^−1^, a concentration that doubled after 5 min of the application of an upper lip twitch [60]. Among them, Lagerweij et al. [61] suggested that despite the fact that the twitch is popularly believed to work by distracting the horse, in fact it has an analogous use to acupuncture, as its actions trigger the release of endorphins from the brain which translates to a calming effect.

McDonnell [62] and later supported by Henderson [63] found remarkable interindividual variation in the time intervals to adapt to the twitch. The same author stated that for most horses that are not upset, it normally takes a few minutes of rest after removing the twitch before reapplication can achieve a worthwhile effect. In a second round, after 10 to 15 min of rest, reapplication resulted in a reasonable rise in endorphins once again, along with the corresponding behavior (droopy lip, glassy eye, and relaxed facial expression). It is helpful to monitor each individual’s attitude. Once signs that effectiveness may be waning are noticed, soothe the horse, gently remove the twitch, massage the lip, offer a treat, and allow the horse to rest for a while. On the second application (after a 10–15 min break), the twitch actions’ effectiveness delays by a minute or so, and as such effectiveness is shorter. McDonnell [62] also suggests that it takes endorphins ~3–5 min to reach reasonable levels so as to produce an effect, which agrees with other authors [60]. The interval from first application to effectiveness is very similar to the response to a standard intravenous (IV) dose of the sedative xylazine. As with the usual sedation approach, it is best to wait until it takes effect to apply the procedure. The typical duration of effectiveness of the first round of twitch application is also similar to that of IV xylazine (~12–15 min, wide interindividual variation in horses). Furthermore, some intraindividual variation from one scenario to another could be expected. Many situational factors may modify the twitch’s effectiveness. Among these factors are the skill, care and respect with which the handler applies the twitch, how well the horse handles the application, how painful the horse may be, how disturbing or uncomfortable the particular procedure and the situation is for that individual, and how calmly and reassuringly the people involved behave. Hence, McDonnel [62] concluded that rather than having guidelines or rule-of-thumb expectations for their application, the twitch handler should continually monitor the animal’s behavior for signs of ineffectiveness (eye, lip, overall facial expression, and muscle relaxation) and the person performing the procedure should respect the advices provided by the handler.

Interspecific variation in the release level of endorphins has been suggested when donkeys were assessed [64]. Vreeman et al. [65] reported a lower response to painful stimuli under influence of the twitch compared to a control group in donkeys, which may be even more remarkable given the stoicism of these species. Mean heart rate (HR) was significantly increased during the twitch procedure after applying the twitch. Administration of painful stimuli during the twitch procedure did not further increase mean HR, while mean HR during the procedure without the twitch significantly increased after applying painful stimuli. Simultaneously, the use of the twitch resulted in a significant increase in mean plasma ACTH concentration. Hence, the use of the twitch did not result in significant differences in mean plasma β-endorphin concentrations. These authors concluded that twitching of donkeys may lead to a hormonal stress response without concurrent increases in ß-endorphin concentration, however the response to painful stimuli was clearly less, which may stem from the behavioral differences between species. Therefore, the use of a twitch for restraining donkeys should be limited to perform mildly painful and/or brief procedures, but the underlying mechanisms may differ from one species to another, which could also be expected in mules and hinnies.

Andrade et al. [64] concluded that Brahman cattle habituated to repeat handling in a squeeze chute were less emotionally reactive when their eyes were covered during this process and established a repeatable order of entrance to the chute. These authors supported their conclusions with the basics provided by Grandin [65], as placing animals in dark stalls reduced the negative impact of handling and restraint on routine reproductive procedures such as artificial insemination. The success of this practice is based on a combination of factors, such as blocking the view of an escape route, preventing the animal from seeing people that are inside its flight zone and utilizing the calming effect produced by darkness [65].

Another form of restraint that may be effective but must be done by a trained individual is lifting and tying the opposite leg with a safety knot held to a slip-proof loop made around the neck [56]. Pharmacological restraint can decrease the level of fear and resistance for routine procedures. Sedation with α2-adrenoreceptor agonists (xylazine and detomidine) alone or in combination with opioids (e.g., butorphanol) can be used as ancillary chemical restraint methods. Detomidine oral gel may also be useful for mules that are difficult to obtain intramuscular or intravenous access [39]. Often, analgesic agents may be metabolized at a higher rate and have to be re-administered to produce the same effect in mules [39].

## 3. Conclusions

A currently limited amount of published information on the behavior of mules and hinnies creates challenges for informing behavior change. These challenges are directly related to the mistreatment and misunderstanding that these hybrids face. This review compiles the little information available which can provide practitioners, handlers, and owners with the key points for interacting with these animals in a positive manner and providing advanced health and husbandry care. Human contact and the human–animal bond, even from early development stages, play a crucial role as early foal handling can promote more gentle and trainable mules and hinnies. Moreover, desensitizing mules to having their ears touched, placing and removing a halter/head collar, picking up hooves and holding them, and taking their temperature may lead to ease of handling at older ages. The assessment of related literature highlights the knowledge gap concerning scientific papers, which has been suggested to be an indirect promotor of equine welfare in donkeys and their hybrids. This review not only addresses the ways to approach the behavior of mules and hinnies, but also suggests research niches that should be further explored in order to make the sound and stastically significant information available regarding handling methods, behavioral signs, restraining procedures, and physiologic indicators, among others. Improved welfare for mules and hinnies is important given the importance of the human–animal bond;once the bond is present, one should work with the animal’s behavior and not against it. Mules are intelligent, not naturally aggressive, and positive interactions can improve behavior and ultimately, welfare. Comparing and contrasting literature and research can lead to the development of a teaching tool in the form of a husbandry guide for handlers of mules and hinnies as a way to counteract the poorly based misconceptions about these hybrids. An educational guide may act as an initial step to increase owner education and promote the overall welfare of mules and hinnies. Future studies should continue to evaluate and quantify mule and hinny behavior. Among the literature gaps addressed, developing a separate mule and hinny ethogram, measuring indicators of stress when these animals are handled or restrained, and measuring the impact of handling foals on eventual mule or hinny behavior are topicswhich may benefit people in direct contact with mules and hinnies the most.

## Supplementary Materials

The following are available online at https://www.mdpi.com/2076-2615/9/8/488/s1, Video S1: Early foal handling with mule foals may improve the ability to perform routine tasks when the mule or hinny ages, as well as improve acceptance of unfamiliar people interacting with them.
